# Mucinous Adenocarcinoma of the Rectum: A Whole Genome Sequencing Study

**DOI:** 10.3389/fonc.2020.01682

**Published:** 2020-08-26

**Authors:** Ian S. Reynolds, Valentina Thomas, Emer O’Connell, Michael Fichtner, Deborah A. McNamara, Elaine W. Kay, Jochen H. M. Prehn, John P. Burke, Simon J. Furney

**Affiliations:** ^1^Department of Colorectal Surgery, Beaumont Hospital, Dublin, Ireland; ^2^Department of Physiology and Medical Physics, Royal College of Surgeons in Ireland, Dublin, Ireland; ^3^Centre for Systems Medicine, Royal College of Surgeons in Ireland, Dublin, Ireland; ^4^Genomic Oncology Research Group, Royal College of Surgeons in Ireland, Dublin, Ireland; ^5^Department of Surgery, Royal College of Surgeons in Ireland, Dublin, Ireland; ^6^Department of Pathology, Beaumont Hospital, Dublin, Ireland

**Keywords:** mucinous adenocarcinoma, rectal cancer, whole genome sequence, genomics, colorectal cancer

## Abstract

**Introduction:**

Mucinous adenocarcinoma of the rectum is an infrequently encountered histological subtype that is associated with an impaired response to chemoradiotherapy and a worse overall prognosis. A genomic profile analysis of mucinous rectal tumors has not yet been performed. The aim of this study was to comprehensively describe the burden of somatic mutations and copy number variation as well as perform mutational signature and microbial analysis of an in-house collected cohort of mucinous adenocarcinoma of the rectum.

**Methods:**

Genomic DNA was extracted from 10 cases of mucinous rectal cancer and matched normal tissue. Whole genome sequencing (WGS) was carried out on these 10 cases and a comprehensive bioinformatic analysis was undertaken.

**Results:**

The average number of SNVs, InDels and SVs in the cohort was 16,600, 1,855, and 120, respectively. A single case was MSI-H. *KRAS* mutations were found in 70% of cases while *TP53* was mutated in only 40% of cases. CNA gain was identified on chromosomes 7, 8, 12, 13, and 20 while CNA loss was found on chromosomes 4, 8, 17, and 18 corresponding to oncogenes and tumor suppressor genes, respectively. Overall mucinous rectal cancers are more likely to be MSI-H and to have *KRAS*, *BRAF*, and *PIK3CA* mutations when compared to rectal adenocarcinoma NOS. Microbial analysis demonstrated an abundance of *Fusobacterium nucleatum* in tumor samples compared to normal tissue.

**Conclusion:**

This study provides a detailed WGS analysis of 10 cases of mucinous rectal cancer. It demonstrates an important lesson in tumor biology in that histologically similar tumors can have extensive differences at the genomic level. This study is relevant as it raises important questions about the relationship between bacteria and malignancy.

## Introduction

Rectal cancer is a frequently encountered malignancy with 704,376 cases recorded in humans worldwide in 2018 and 310,394 deaths from the disease in the same year, it is most prevalent in Western Countries (1). Approximately 28% of all colorectal cancers (CRCs) are located in the rectum with some variation observed between males and females in that 32% of CRCs are found in the rectum in males compared to 25% in females (2). Neoadjuvant chemoradiotherapy using a fluoropyrimidine agent followed by total mesorectal excision (TME) remains the treatment of choice for the majority of rectal cancers (3–5). The aims of neoadjuvant chemoradiotherapy are to downstage the primary tumor, reduce margin positivity rates, decrease local recurrence and increase sphincter preservation rates. Response to treatment is variable with pathological complete response (pCR) rates ranging from 4 to 30% and tumor downstaging occurring in up to 45% of patients (6, 7). Identifying those patients that are going to have a poor response to chemoradiotherapy prior to commencing treatment would clearly be advantageous as they could potentially be offered alternative treatments possibly using targeted therapies (8).

Adenocarcinoma not otherwise specified (NOS) is the most frequent histological subtype encountered followed by mucinous adenocarcinoma which accounts for approximately 10% of all rectal cancers (9, 10). A tumor is defined as mucinous adenocarcinoma when more than 50% of the lesion is composed of pools of extracellular mucin. Mucinous adenocarcinoma of the rectum has been shown to be associated with a poor response to neoadjuvant chemoradiotherapy resulting in a reduced rate of pCR, reduced tumor downstaging, increased margin positivity and poorer overall survival (11). The reasons underpinning the poor response to treatment remain poorly understood and may be related to a physical barrier formed by mucin production, however, we hypothesize that they more likely relate to distinct genomic aberrations and alternative intracellular signaling mechanisms when compared to the adenocarcinoma NOS subtype. Previous studies have shown that mucinous CRCs are more likely to harbor mutations in *KRAS* and *BRAF* and that they are more likely be associated with microsatellite instability (MSI) and the CpG island methylator phenotype (CIMP) (12), however, the bulk of this data pertains to colonic tumors and little is known about the true molecular association of mucinous adenocarcinoma of the rectum. The aim of this study was to define the genomic landscape of mucinous adenocarcinoma of the rectum using whole genome sequencing (WGS).

## Materials and Methods

### Patient Cohort

Our prospectively maintained board approved rectal cancer database was interrogated to identify cases of mucinous adenocarcinoma of the rectum treated in our institution in the 10-year period between January 1st 2008 and December 31st 2017. All cases had to meet the World Health Organization (WHO) criteria for mucinous adenocarcinoma in that more than 50% of the lesion had to be composed of pools of extracellular mucin (13). The diagnosis of mucinous adenocarcinoma was based on the pre-treatment biopsy specimen as mucin seen in the resection specimen may have been due to the effects of treatment. The Mandard tumor regression grading score was used to assess response to neoadjuvant chemoradiotherapy (14). For all cases identified we performed a cross check with our biobank to ensure that there was fresh frozen tissue available for both normal rectum and tumor. All fresh frozen samples were checked by a consultant pathologist by taking small sections and staining them with hematoxylin and eosin (H&E) to confirm that they were normal or tumor and also to ensure that the tumor was in-fact mucinous adenocarcinoma. Any cases that did not have adequate fresh frozen normal or tumor tissue available were excluded. In our institution all new rectal cancer cases are consented for inclusion in our biobank for future studies and hence we often have fresh frozen samples from the biopsy and resection available for most patients. Ethical approval for this study was granted by the Beaumont Hospital Research Ethics Committee (REC reference 18/11).

### Sample Preparation

Genomic deoxyribonucleic acid (gDNA) was extracted from all tissues by following the manufacturer’s instructions of the Qiagen AllPrep DNA/RNA/Protein Mini Kit (see Supplementary Material and Methods for full description of DNA extraction). The sample was quantified by following the manufacturer’s instructions on the Qubit dsDNA HS Assay Kit. The quality of the extracted DNA was evaluated by agarose gel electrophoresis. A 1.0% agarose gel was prepared. 200 ng of DNA in 10 μL was mixed with 2 μL of 6x loading dye. The gel was run at 100 volts for 40 min and then evaluated using ultraviolet (UV) light to look for any evidence of DNA degradation.

### Whole-Genome Sequencing

One sample of the primary tumor and one of normal tissue were collected for WGS from each patient. For the majority of patients 1 μg of genomic DNA at a minimum concentration of 12.5 ng/μL was prepared. A low DNA input option was used for normal and tumor in one case and for normal only in a second case as there was an insufficient quantity of DNA for the regular input option, for these cases at least 200 ng of genomic DNA at a minimum concentration of 2.5 ng/μL was prepared. Prior to shipping the samples were prepared to the desired concentration using elution buffer. The samples were then shipped to Beijing Genomics Institute (BGI) in Hong Kong on dry ice. Library construction was carried out in BGI and included the following steps; fragmentation, size selection, end repair and A-tailing, adaptor ligation and polymerase chain reaction (PCR) and finally splint circularization. Paired end sequencing reads (151 bp) were generated using BGISEQ sequencing technology, yielding ∼60 × coverage for the tumor samples and ∼30 × coverage for the normal samples. Sequences were aligned to the human reference genome (GRCh37) using BWA (15) and PCR duplicates were marked using Picard Tools^1^.

### Mutation Discovery: Substitutions, InDels, and Structural Variants

Somatic mutations were identified by comparing each tumor sample with adjacent healthy rectal tissue as a matched normal. Single nucleotide variants (SNVs) were identified with the mutation calling algorithm Strelka v1 (16) and annotated with the Ensembl Variant Effect Predictor (VEP) v97 (17). Variants with a dbSNP identifier^2^ were removed and variants with a COSMIC identifier^3^ were retained. We calculated the variant allele frequency (VAF) of each SNV and further validated mutations by only keeping the ones that met the following parameters: normal alternate allele < = 1, minimum combined depth = 20, minimum alternate depth = 2, minimum VAF = 0.05. Insertion deletion mutations (InDels) were identified with the mutation calling algorithm Strelka v1 (16) and annotated with the Ensembl Variant Effect Predictor (VEP) v97 (17). Variants with a dbSNP identifier (see text footnote 2) were removed and variants with a COSMIC identifier (see text footnote 3) were retained. Structural variants (SVs) were identified using DELLY v0.7.9 (18).

### Copy Number Alterations

Copy number alterations (CNAs) were identified using the R package FACETS v0.5.14 (19) and visualized with the R package copynumber v1.24.0 (20).

### Gene Annotation and Driver Analysis

The most frequently mutated genes were identified using the R package maftools v2.2.10 (21). The genic location and functional impact of SNVs, InDels and SVs was annotated using the Ensembl Variant Effect Predictor (VEP) v97 (17). Known driver genes, together with MMR genes and genes coding for mucin glycoproteins, were searched for causative mutations in all samples. The VAF of each identified driver was calculated to establish its prevalence. CNAs were annotated using the annotate_variation function implemented by ANNOVAR v2019Oct24 (22) and searched for drivers based on known CRC-associated somatic gene copy number alterations (23). The relevance of each putative driver CNA was estimated through its median log-ratio, which was provided by the FACETS analysis (19).

### Mutational Signature Analysis

Mutational signature analysis was performed to inform on the exposures and biological history of a cancer. Mutational signatures were identified from SNVs using the R package deconstructSigs v1.9 (24) based on the pan-cancer catalog of single base substitution (SBS) signatures referenced in the COSMIC v3 database^4^.

### Gut Microbiome Analysis

Tumor and normal tissue-associated gut microbiota were identified from DNA data using PathSeq v2.0 (25), available from the Genome Analysis Toolkit (GATK) v4^5^.

### Comparison to Rectal Adenocarcinoma NOS

Mutations and CNA in clinically relevant and well known genes in the mucinous rectal cases from the Beaumont cohort as well as the mucinous rectal cases from The Cancer Genome Atlas (TCGA) cohort were compared to the rectal adenocarcinoma NOS specified cases from TCGA. The frequency of mutations in the following genes were compared; *KRAS*, *BRAF*, *PIK3CA*, *TP53*, and *APC.* Copy number alterations were compared in the following genes *KRAS*, *TP53*, *APC*, *EGFR*, and *MYC.* Comparison was performed using Fisher’s exact test on GraphPad Prism version 8.3.0. To enable comparison of tumor mutational burden (TMB) between the WGS data and TCGA exome data, TMB estimation was conducted using mutations identified in CCDS (Consensus CDS) exons only.

## Results

### Patient Cohort

To investigate the genomic landscape of mucinous adenocarcinoma of the rectum the whole genomes of 10 mucinous rectal cancers were sequenced along with their matched germline genomes. The cohort included 6 males and 4 females. Neoadjuvant chemoradiotherapy was utilized in 6 of the 10 patients. Sequencing was performed on the biopsy specimen in 5 cases and in the resection specimen in the remaining 5 cases. Neoadjuvant chemoradiotherapy was utilized in only 2 of the 5 cases where the resection specimen was sequenced, this meant that 8 out of the 10 samples that were sequenced were not exposed to the effects of chemoradiotherapy while 2 samples were (Cases H and I). When the resection specimens underwent pathological staging a single patient had stage I disease, 3 patients had stage II disease and 6 patients had stage III disease. A Mandard tumor regression grade (TRG) 3 was found in two patients, a TRG 4 was found in three patients and a TRG5 5 was identified in a single patients (see Table 1).

**TABLE 1:** Baseline Patient Characteristics.

	Age group	Stage	pT Stage	pN Stage	Neoadjuvant CRT	TRG	Source
Case A	51–55	1	2	0	No	N/A	Biopsy
Case B	66–70	3	3	1	Yes	TRG5	Biopsy
Case C	71–75	2	3	0	Yes	TRG4	Biopsy
Case D	76–80	3	3	1	Yes	TRG4	Biopsy
Case E	66–70	3	3	1	Yes	TRG3	Biopsy
Case F	71–75	3	3	2	No	N/A	Resection
Case G	71–75	3	2	1	No	N/A	Resection
Case H	26–30	2	3	0	Yes	TRG3	Resection
Case I	81–85	2	3	0	Yes	TRG4	Resection
Case J	56–60	3	4	2	No	N/A	Resection

*CRT, chemoradiotherapy; TRG, tumor regression grade.*

### Mutational Load and Driver Analysis of SNVs, InDels, and SVs

**FIGURE 1:**
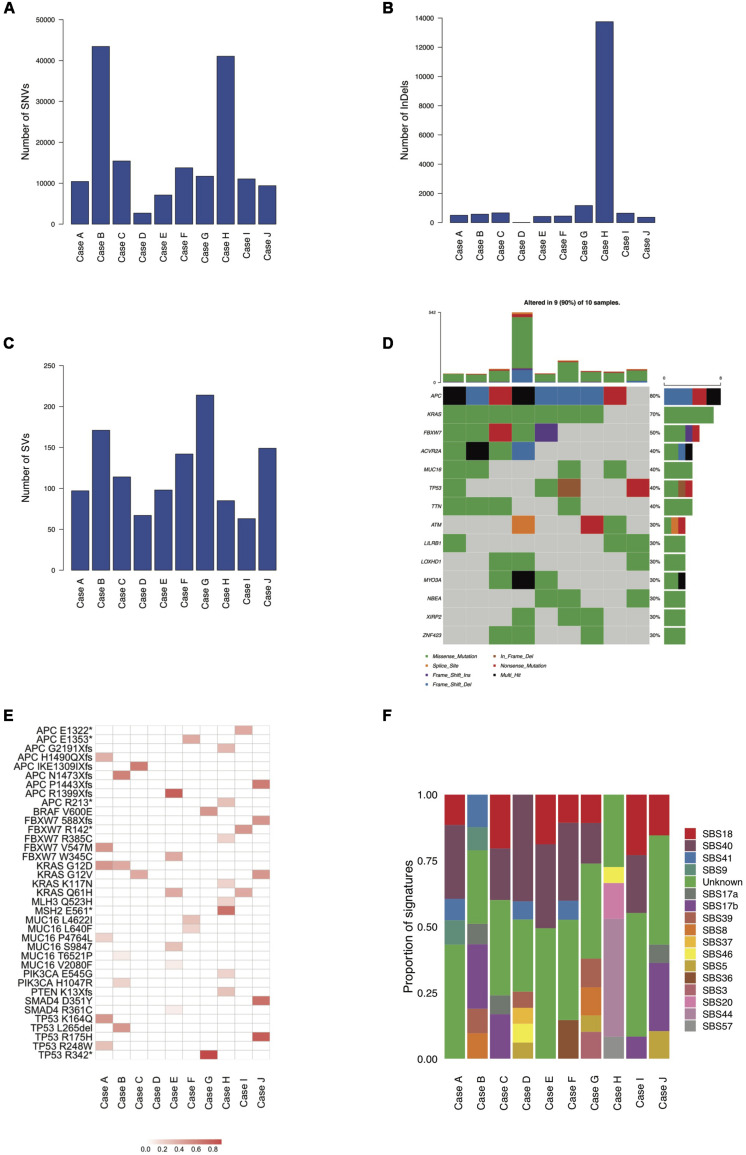
Mutational burdens, CRC driver genes and mutational signatures in the ten mucinous rectal cancer genomes (Cases A, B, C, D, E, F, G, H, I, and J). Mutational burdens of SNVs **(A)**, InDels **(B)** and SVs **(C)** quantified by whole-genome sequencing. **(D)** Most frequently mutated genes (30% of samples and above). **(E)** VAFs of CRC-associated driver genes. **(F)** Tumors mutational profiles of total SNVs, defined by the weighted contributions of each input reference signature from COSMIC and identified by whole-genome sequencing.

We identified an average of 16,600 SNVs, 1,855 InDels, and 120 SVs per tumor. The lowest record of SNVs (2,691) was seen in case D while the highest values were reported in cases B and H (43,430 and 41,039, respectively), where SNV occurrence was therefore at least 2.6 times that of the rest of the samples (see Figure 1A). InDel abundance peaked in case H (13,747), where incidence was at least 11.7 times that of all other cases (range: 12 to 1,168; see Figure 1B). SVs were the least common variant type, ranging from 63 in case I to 214 in case G, with ∼3.4-fold frequency variance between the two (see Figure 1C). The genes most frequently affected by putative deleterious somatic mutations were *APC* (mutated in 80% of samples), *KRAS* (mutated in 70% of samples), *FBXW7* (mutated in 50% of samples), *ACVR2A* (mutated in 40% of samples), *MUC16* (mutated in 40% of samples), *TP53* (mutated in 40% of samples) and *TTN* (mutated in 40% of samples). With the exception of case D, all tumors contained likely functional mutations in known CRC genes. Most mutations were unique to each cancer sample. Somatic mutations in the mismatch repair (MMR) genes *MLH3* and *MSH2* were seen only in the MSI case (case H) which also had a germline mutation in *MSH2*. The mutation found was a G to T mutation in exon 11 of the *MSH2* gene and resulted in a stop gained high impact mutation. The *BRAF* V600E mutation occurred only in case G and a higher mutational burden did not correlate with a greater number of putative deleterious mutations (see Figures 1D,E).

### Mutational Signatures

Mutational signature analysis (see text footnote 4) shows broadly similar mutational profiles for most cases with the exception of case H, for which a distinct mutational profile emerged, characterized by a significant proportion of the MMR-deficiency related signatures SBS20 and SBS44, which are absent in all other samples. Most of the signatures reported are of unknown etiology (SBS40, SBS41, SBS17a, SBS17b, SBS39, SBS8, and SBS37) while two are possible sequencing artifacts (SBS46 and SBS57). SBS18 occurs in most samples and is possibly due to damage by reactive oxygen species. Signature SBS9, attributed to polymerase η, is found in cases A and B, while the age-related SBS5 signature is only detected in cases D, G, and J. SBS36, possibly linked to defective base excision repair, is found in case F. Evidence of the HR-deficiency related signature SBS3 is found in case G (see Figure 1F).

### Copy Number Alterations and CNA Driver Analysis

**FIGURE 2:**
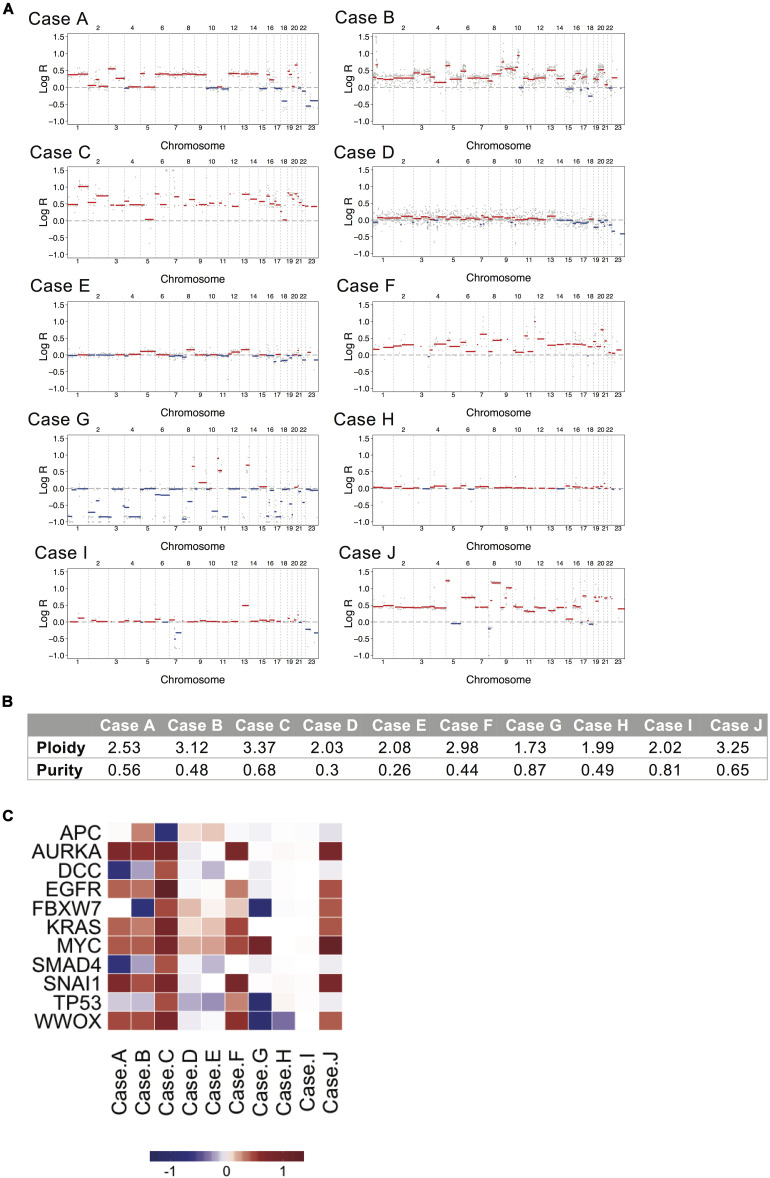
Copy number alteration analysis for the ten mucinous rectal cancer genomes. **(A)** The genome landscapes show copy number alterations estimated by whole-genome sequencing for Cases A, B, C, D, E, F, G, H, I, and J. The *x*-axis coordinates represent positions along the genome, with vertical bars indicating the borders between chromosomes. The *y*-axis represents the chromosomal copy number log-ratio in the tumor. Amplifications are depicted in red and deletions in blue. **(B)** Values of tumor ploidy and tumor purity, as estimated by FACETS. **(C)** Median log-ratio of putative driver CNAs. Positive log-ratios (gains) are represented in red and negative log-rations (losses) are represented in blue.

Somatic copy number analysis revealed variation in chromosomal instability (CIN) in the 10 samples (see Figures 2A,B and Supplementary Figure S1). Cases A, B, C, F, and J showed hyperdiploid genomes (ploidy: 2.53, 3.12, 3.37, 2.98, and 3.25, respectively), while case G showed a hypodiploid one (ploidy: 1.73). A stable genome was observed in cases D, E, H, and I (ploidy values ranging from 1.99 to 2.08). Regional genomic alterations typical of CRC were observed, such as gains at chromosome 7 and 8 (cases A, B, C, F, and J), 12 and 13 (cases A, B, C, F, I, and J), 20 (cases A, B, C, F, and J) and losses at chromosome 4 and 8 (case G), and 17 and 18 (cases A, B, E, and G). Oncogenes such as *EGFR*, *MYC*, *KRAS*, *SNAI1* and *AURKA* were consistently amplified in hyperdiploid cases A, B, C, F and J. *MYC* was also highly amplified in case G. Deletions occurred in tumor suppressor genes such as *DCC*, *SMAD4*, *FBXW7*, *APC*, *TP53*, and *WWOX* in cases A, B, C, G, and H (see Figure 2C).

### Microbial Analysis

**FIGURE 3:**
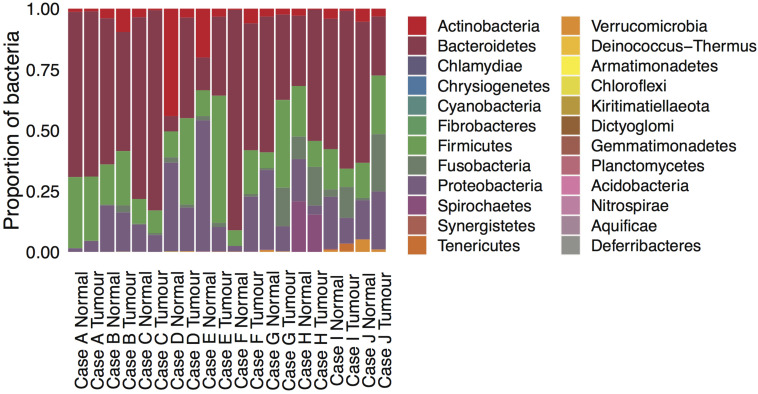
Microbial composition in the ten mucinous rectal cancer genomes. Normalized scores of bacterial abundances at the phylum taxonomic level, as estimated by whole-genome sequencing for Cases A, B, C, D, E, F, G, H, I, and J and their respective normal tissue.

DNA analysis of gut microbial organisms associated with each tumor and normal sample in cases A-J revealed prevalence of *Bacteroidetes*, *Firmicutes*, *Proteobacteria*, and *Actinobacteria* species across all samples. Evidence of *Fusobacteria* spp. was detected in all cases but their abundance varied across samples, being generally greater in the tumor than in its matched normal tissue (see Supplementary Table S1). The *Fusobacterium nucleatum* species was detected in all samples except case A Normal, with the highest occurrences in tumor cases I and J (see Supplementary Table S2). Significantly higher prevalence of *Spirochetes* spp. was found in the tumor and normal samples of case H (see Figure 3).

### Comparison to Rectal Adenocarcinoma NOS

**FIGURE 4:**
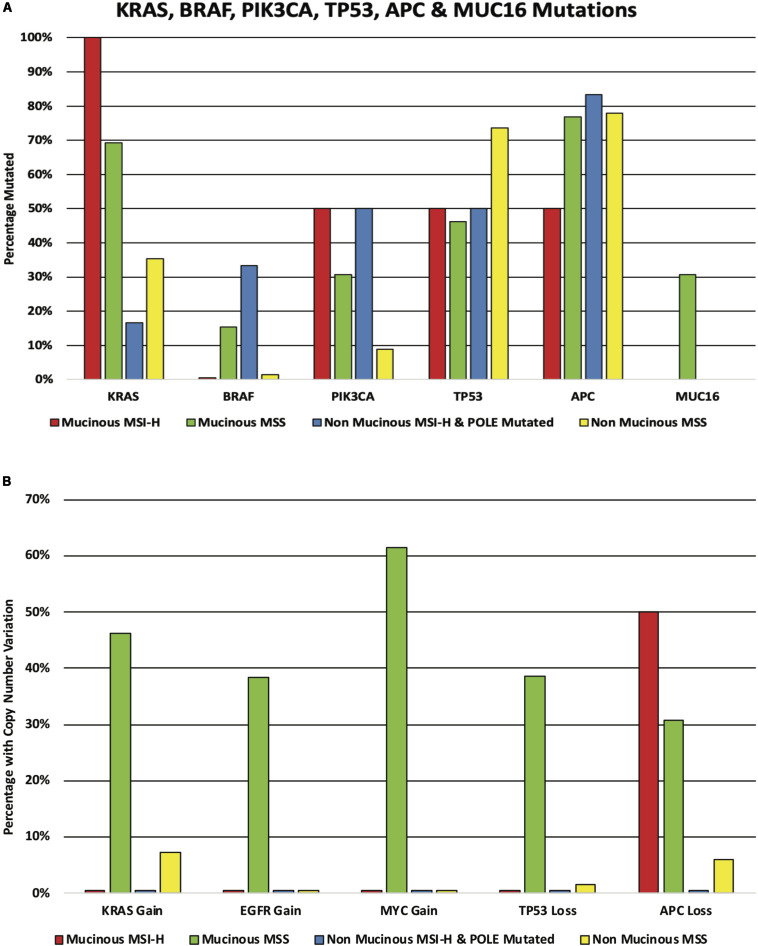
**(A)** KRAS, BRAF, PIK3CA, TP53, APC, and MUC16 mutations in the mucinous and non-mucinous cohorts. The cohorts are subdivided according to mutator status. **(B)** KRAS, EGFR, MYC, TP53, and APC copy number alteration in the mucinous and non-mucinous cohorts. The cohorts are subdivided according to mutator status.

The results shown below are in part based upon data generated by the TCGA Research Network^6^. There were a further 5 cases of mucinous rectal adenocarcinoma in the TCGA cohort bringing the total number of mucinous cases to 15. These 15 cases were compared to 74 cases of adenocarcinoma NOS. The cohorts were subdivided into two groups; microsatellite stable (MSS) and hypermutator (MSI-H and *POLE* mutated). The mucinous group had 2 hypermutator patients (both MSI-H) out of the cohort of 15 patients while the adenocarcinoma NOS specified group had 6 hypermutator patients (2 MSI-H and 4 *POLE* mutated) out of the cohort of 74 patients. *KRAS* mutations were found in 100% of the mucinous hypermutator patients compared to just 16.7% of adenocarcinoma NOS hypermutator patients (*p* = 0.11). The *KRAS* mutation rate was 69.2% versus 35.3% in the mucinous and adenocarcinoma NOS MSS cohorts, respectively (*p* = 0.03). *NRAS* was mutated in 6.8% of the adenocarcinoma NOS cohort compared to 0.0% of the mucinous cohort (*p* = 0.58). *BRAF* mutations were found in 15.4% of the mucinous MSS cohort compared to 1.5% of the adenocarcinoma NOS MSS cohort (*p* = 0.07). *PIK3CA* mutations were more common in the mucinous MSS cohort compared to the adenocarcinoma NOS MSS cohort (30.8% vs. 8.8% *p* < 0.05). No differences were identified in the frequency of *TP53* or *APC* mutations between the cohorts. *MUC16* mutations were more frequent in the mucinous MSS cohort compared to the non-mucinous MSS cohort (30.8% vs. 0.0%, *p* < 0.001) (see Figure 4A). There were no *MUC16* mutations in either of the MSI cohorts. CNA gain of *KRAS* was more common in the mucinous MSS cohort compared to the adenocarcinoma NOS MSS cohort (46.4% vs. 7.4% *p* = 0.002). CNA gain of *EGRF* was also more common in the mucinous MSS cohort compared to the adenocarcinoma NOS MSS cohort (38.5% vs. 0.0% *p* < 0.0001) as was CNA gain of *MYC* (61.5% vs. 0.0% *p* < 0.0001). CNA loss of *TP53* was more common in the mucinous MSS cohort compared to the adenocarcinoma NOS cohort (38.6% vs. 1.47% *p* = 0.0003) as was CNA loss of *APC* (30.8% vs. 5.9% *p* = 0.02) (see Figure 4B). A comparison of the median number of mutations per megabase demonstrated that tumor mutational burden (TMB) was higher in the adenocarcinoma NOS MSS cohort when compared to the mucinous MSS cohort (3.61 vs. 2.76 mutations per Mb; *p* = 0.03). The median number of mutations per megabase in the adenocarcinoma NOS hypermutator and mucinous hypermutator cohorts were 82.19 and 28.18 mutations per Mb, respectively (*p* = 0.33).

## Discussion

In this study, we present the first WGS data analysis of mucinous adenocarcinoma of the rectum. We have elucidated the mutational load of SNVs, InDels and structural variants, we described mutational signatures, CNA load and driver analysis as well as microbial abundance. We have demonstrated how a group of morphologically similar tumors at the microscopic level show major differences at the genomic level. Overall this cohort had an impaired response to neoadjuvant chemoradiotherapy when compared to adenocarcinoma NOS (11). This is in line with previous studies, however, it is known that some patients with mucinous rectal cancer do respond to chemoradiotherapy (26–29). Furthermore, there is evidence to suggest that some mucinous tumors might overexpress genes or have somatic mutations in genes encoding for enzymes involved in the metabolism of pyrimidine and platinum-based compounds and this may result in chemoresistance (30, 31).

The most frequently mutated genes in the Beaumont cohort included; *APC* (80%), *KRAS* (70%) *FBXW7* (50%), *TP53* (40%), and *MUC16* (40%). When pooled together with cases from TCGA the mutational frequency is different for many of these genes in comparison to the rectal adenocarcinoma NOS specified cohort. In particular *KRAS* and *PIK3CA* mutations were more common in the mucinous cohort. CNA gain in *KRAS*, *EGFR* and *MYC* was more common in the mucinous cohort as was CNA loss in *TP53* and *APC*. There was one MSI-H case in the Beaumont cohort and one in the TCGA mucinous cohort making the mucinous MSI-H rate 13.3% compared to 2.7% in the TCGA rectal adenocarcinoma NOS cohort. While the cohort is small the MSI-H rate of 13.3% closely mirrors results of unpublished work from our research group in a larger cohort of 33 mucinous rectal cancers where the MMR-deficiency rate was found to be 12.1%. Two mucinous cases had a mutation in the *BRAF* gene again representing 13.3% of the cohort compared to 4.1% of the TCGA adenocarcinoma NOS cohort. Although the numbers of mucinous cases are small, the higher frequency of MSI-H and *BRAF* mutations in the mucinous rectal cohort is in keeping with what is already known about mucinous colon tumors (12). *MUC16* mutations were found in 26.7% of the mucinous rectal cases and one of the MSI-H mucinous cases also had an SNV in *MUC21.* The only mutations found in mucin genes in the TCGA rectal adenocarcinoma NOS cohort was a 9.5% mutation rate in the *MUC1* gene. The significance of mutations in mucin glycoprotein genes is not known and our current understanding is that the mucinous phenotype is the result of increased expression of the *MUC2* gene (32–34). The increased frequency of CNA loss of *APC* and *TP53* mutations in the mucinous cohort is again reflective of what is found in mucinous colon tumors and likely represents the development and progression of some of these tumors along pathways other than the traditional CIN pathway (35). The mutational signature analysis revealed many signatures of unknown etiology across the samples (i.e., SBS40, SBS41, SBS17a, SBS17b, SBS39, SBS8, and SBS37). SBS9 which is attributed to polymerase η and is traditionally associated with skin cancers, leukemia and lymphoma was found in cases A and B (36). A single case (Case G) had evidence of SBS3 which is associated with failure of DNA double-strand break-repair by homologous recombination and is associated with both germline and somatic mutations in *BRCA1* and *BRCA2*, however, we found only a single *BRCA2* mutation in our cohort in case B. This signature is associated with sensitivity to platinum based chemotherapy and PARP inhibitors. As expected the MSI-H case (case H) showed evidence of signatures SBS20 and SBS44, the MMR-deficiency related signatures. These mutational signatures are associated with defective DNA MMR and are caused by high numbers of insertions and deletions and poly and mononucleotide repeats.

The microbial analysis of the 10 cases revealed the presence of *Bacteroidetes*, *Firmicutes*, *Proteobacteria*, and *Actinobacteria* species across all samples. Of particular interest was the presence of *Fusobacteria* species detected in all cases, and specifically the presence of *F. nucleatum* detected in all samples except the sample of normal tissue from case A. The abundance of *Fusobacteria* was generally greater in the tumor samples compared to the normal samples in our cohort. A study by Dharmani et al. has demonstrated how *F. nucleatum* infection of colonic cells can stimulate the production of *MUC2* mucin and tumor necrosis factor alpha (TNFα) through increased gene expression (37). Increased expression of *MUC2* is found in most mucinous CRCs and the increased abundance of *F. nucleatum* may play some part in the differentiation of tumors into the mucinous subtype (32, 34, 38, 39). *F. nucleatum* has also been shown to be capable of promoting the development of malignancy from inflammation by causing oxidative stress to epithelial and stromal cells which results in DNA damage (37, 40–43). Furthermore, *F. nucleatum* is known to be associated with cancers that are MSI-H, cancers with *MLH1* hypermethylation, CpG island methylator phenotype (CIMP) cancers and cancers that have a poor prognosis (44). Flanagan et al. demonstrated how *F. nucleatum* levels are more abundant in colorectal adenocarcinoma NOS tissue compared to match normal tissue, they also showed that patients with low levels of the bacterium have significantly longer overall survival when compared to patients with moderate and high levels (45). The proportion of *F. nucleatum* high cases in CRCs gradually increases from rectum to cecum, similar to mucinous histology. This makes our finding of abundant amounts of *F. nucleatum* in the majority of our samples a pertinent finding and supports the association between *F. nucleatum* and mucinous CRCs (46–48). *F. nucleatum* has also been shown to promote chemoresistance in colorectal cancer by modulating autophagy (49) and this raises an important question as to whether outcomes for patients with CRC can be improved by eradication of *F. nucleatum.* Bullman et al. have demonstrated that treatment with metronidazole of *Fusobacteria-*colonized patient derived xenografts (PDXs) in mice reduced tumor growth and proliferation *in vivo* as well as decreasing the *Fusobacterium* load (50). Clearly there is scope for further research focusing on the effects of reducing *Fusobacterium* loads in patients with mucinous CRC to identify any potential relationship with antimicrobial therapy and response to chemoradiotherapy as well as prognosis.

The rapidly increasing number of genomes that are being sequenced is helping to identify the molecular mechanisms at play in a wide array of cancers and benign diseases (51). It is now apparent that cancers that appear morphologically similar are often vastly different at the genomic level. These genomic differences influence the behavior of the tumor, its response to treatment and the overall prognosis for the individual patient. The use of sequencing data in clinical practice is still in its infancy, particularly in the setting of CRC where mutations in *RAS* and *BRAF* as well as the MMR/MSI status are the biomarkers that are predominantly used when deciding on adjuvant treatment strategies for patients with metastatic disease. Currently almost all patients undergo the same neoadjuvant and adjuvant treatment protocols as recommended by best practice international guidelines (52–56). It is hoped that the use of next generation sequencing (NGS) will be able to stratify patients into different therapeutic and prognostic groups so that patients will only be offered treatments that they need and that they are likely to respond to. In the future it is possible that extra samples taken at the time of the biopsy may be sent for WGS, whole exome sequencing or targeted gene panel sequencing and combined with gene expression analysis. The results of these studies could be used when deciding whether or not to offer neoadjuvant treatment and also to help decide the optimal treatment regimen to use. For example inactivating mutations and reduced expression of thymidine phosphorylase have been shown to be associated with impaired response to 5-fluorouracil (5-FU) (57) and if these features were identified in a tumor 5-FU could be replaced with an alternative drug to improve the chances of inducing radiosensitivity in the tumor.

The main limitation of this study is the small number of patients that were analyzed and our results will require validation in a larger cohort. The small number of cases is due to the fact that this histological subtype is infrequently encountered in the rectum, furthermore the biobanked samples are often of low quality due to the sheer amount of mucin and relative acellularity of these tumors. In addition, due to the limited amounts of tumor DNA available, and low quantities and quality of extracted RNA, it was not possible to conduct gene expression profiling or other experiments such as methylation analyses. Nevertheless, as far as we are aware, this is currently the largest WGS study of mucinous rectal tumors; at present only 5 cases of mucinous adenocarcinoma of the rectum are included in the TCGA database. A further limitation is the heterogeneity in the tissue source with some samples coming from pre-treatment biopsies and others coming from resection specimens of which two were exposed to chemoradiotherapy (Case H and Case I) as there was no remaining fresh frozen tissue available from the pre-treatment biopsy for sequencing. Both cases showed little evidence of tumor regression in the post-treatment resection specimen and there were no discernible genomic changes related to treatment. A recent publication by Pich et al. highlighted the mutational footprints of several cancer therapies, however, the average exposure to chemotherapy in their cohort was 21 weeks while the two cases in our study received only 6 weeks of chemoradiotherapy (58). Unfortunately there were no matched pre and post treatment samples for any cases so an analysis of the effects of chemoradiotherapy on tumor genomics was not possible.

Going forward we envisage that sequencing of cancer genomes post treatment will likely need to be carried out on tumors from patients who have disease progression while on treatment in order to identify newly acquired somatic mutations and to try and decide which therapeutic strategies might be most effective to deal with the progression.

## Conclusion

Mucinous adenocarcinoma of the rectum is a morphologically distinct subtype that is associated with an impaired response to chemoradiotherapy and a worse prognosis. We present comprehensive genomic profiling of 10 cases and have demonstrated the diverse genomic nature within this histological subtype. Mucinous rectal tumors are more likely to be MSI-H, *BRAF* mutated and *KRAS* mutated which could potentially result in an impaired response to cytotoxic chemotherapy and *EGFR* inhibitors although this is only a hypothesis and needs to be confirmed prospectively with a much larger cohort. They also have an increased frequency of mutations in the *MUC16* gene, however, the significance of this requires further exploration. They also appear to have an abundance of *F. nucleatum*, an anaerobic bacterium that is itself associated with chemoresistance and poor prognosis. Sequencing of the cancer genome from these tumors may allow more appropriate treatment options to be considered up-front and potentially improve outcomes. The potential use of antimicrobial therapy in this cohort needs further exploration.

## Data Availability Statement

The datasets presented in this study can be found in online repositories. The names of the repository/repositories and accession number(s) can be found below: European Genome-Phenome Archive (https://ega-archive.org/ – Project ID: EGAC00001001585).

## Ethics Statement

The studies involving human participants were reviewed and approved by Beaumont Hospital Research Ethics Committee. The patients/participants provided their written informed consent to participate in this study.

## Author Contributions

SF, JB, and JP: study concept and design. IR, EO’C, MF, EK, JB, and SF: study materials. IR: data collection. VT and SF: bioinformatic analysis. IR, VT, and SF: statistical analysis. All authors: manuscript preparation and review.

## Conflict of Interest

The authors declare that the research was conducted in the absence of any commercial or financial relationships that could be construed as a potential conflict of interest.

## Footnotes

1
http://broadinstitute.github.io/picard

2
https://www.ncbi.nlm.nih.gov/SNP/

3
https://cancer.sanger.ac.uk/cosmic

4
https://cancer.sanger.ac.uk/cosmic/signatures

5
http://www.broadinstitute.org/software/pathseq/

6
https://www.cancer.gov/tcga

## Supplementary Material

FIGURE S1 Copy number alterations for the ten mucinous rectal cancer genomes. The genome landscape shows frequency of copy number alterations estimated by whole-genome sequencing for all Cases A, B, C, D, E, F, G, H, I, and J. The *x*-axis coordinates represent positions along the genome, with vertical bars indicating the borders between chromosomes. The *y*-axis represents the percentage of samples with estimated log2 copy number ratios above the threshold 0.05 (gain) in red and below the threshold of −0.05 (loss) in blue.

TABLE S1 Abundance of *Fusobacteria* (phylum level).

TABLE S2 Abundance of *F. nucleatum* (species level).
