# Corrigendum: Mucinous adenocarcinoma of the rectum: a whole genome sequencing study

**DOI:** 10.3389/fonc.2023.1229013

**Published:** 2023-06-28

**Authors:** Ian S. Reynolds, Valentina Thomas, Emer O’Connell, Michael Fichtner, Deborah A. McNamara, Elaine W. Kay, Jochen H. M. Prehn, John P. Burke, Simon J. Furney

**Affiliations:** ^1^ Department of Colorectal Surgery, Beaumont Hospital, Dublin, Ireland; ^2^ Department of Physiology and Medical Physics, Royal College of Surgeons in Ireland, Dublin, Ireland; ^3^ Centre for Systems Medicine, Royal College of Surgeons in Ireland, Dublin, Ireland; ^4^ Genomic Oncology Research Group, Royal College of Surgeons in Ireland, Dublin, Ireland; ^5^ Department of Surgery, Royal College of Surgeons in Ireland, Dublin, Ireland; ^6^ Department of Pathology, Beaumont Hospital, Dublin, Ireland

**Keywords:** mucinous adenocarcinoma, rectal cancer, whole genome sequence, genomics, colorectal cancer

In the published article, there was an error in the Funding statement. The corresponding author’s funding was inadvertently omitted. The correct Funding statement appears below.

“Funding for this project was provided by the Beaumont Hospital Colorectal Research Fund, Science Foundation Ireland (15/ERACSM/3268), and the SFI-HRB-Wellcome Research Partnership (212529/Z/18/Z)”.

The authors apologize for this error and state that this does not change the scientific conclusions of the article in any way. The original article has been updated.

