# Supplementary figures and images for: Mucinous Adenocarcinoma of the Rectum: A Whole Genome Sequencing Study
Source: Front Oncol. 2020 Aug 26;10:1682. doi: 10.3389/fonc.2020.01682 (PMC7479243; doi:10.3389/fonc.2020.01682)

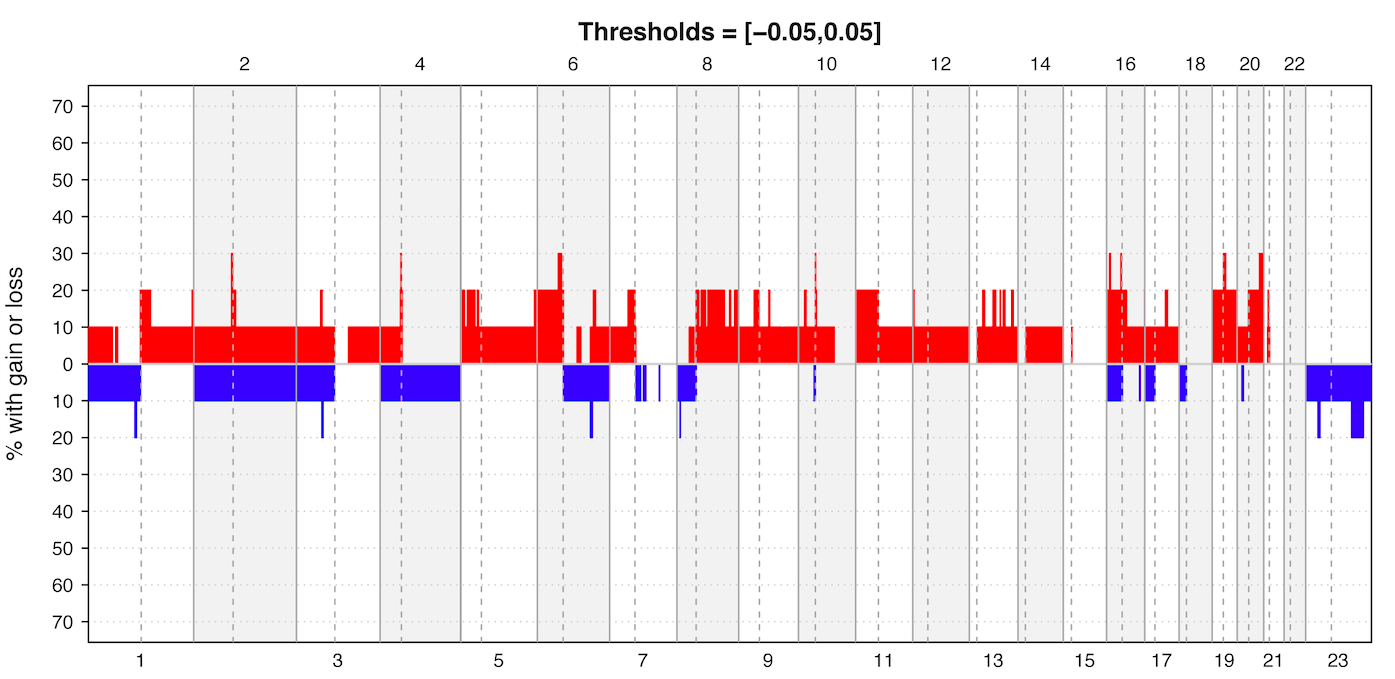

Supplement: FIGURE S1 — Copy number alterations for the ten mucinous rectal cancer genomes. The genome landscape shows frequency of copy number alterations estimated by whole-genome sequencing for all Cases A, B, C, D, E, F, G, H, I, and J. The x-axis coordinates represent positions along the genome, with vertical bars indicating the borders between chromosomes. The y-axis represents the percentage of samples with estimated log2 copy number ratios above the threshold 0.05 (gain) in red and below the threshold of −0.05 (loss) in blue. [file Image_1.png]
