# Supplementary material for: Mucinous Adenocarcinoma of the Rectum: A Whole Genome Sequencing Study
Source: Front Oncol. 2020 Aug 26;10:1682. doi: 10.3389/fonc.2020.01682 (PMC7479243; doi:10.3389/fonc.2020.01682)
Supplement: TABLE S1 — Abundance of Fusobacteria (phylum level). [file Table_1.docx]

|  | **Normal** | **Tumour** |
| --- | --- | --- |
| **Case A** | **0.043592417** | **0.201442144** |
| **Case B** | **0.299837499** | **2.879175916** |
| **Case C** | **0.366427706** | **0.823182797** |
| **Case D** | **2.1829401** | **1.231522339** |
| **Case E** | **1.722902598** | **1.724361316** |
| **Case F** | **0.005849015** | **1.097121872** |
| **Case G** | **0.849104809** | **15.83200083** |
| **Case H** | **9.27234526** | **15.85634849** |
| **Case I** | **3.002054509** | **12.70667967** |
| **Case J** | **1.019829437** | **23.55695037** |

**Supplementary Table 1: Abundance of *Fusobacteria* (phylum level)**
