# Supplementary material for: Mucinous Adenocarcinoma of the Rectum: A Whole Genome Sequencing Study
Source: Front Oncol. 2020 Aug 26;10:1682. doi: 10.3389/fonc.2020.01682 (PMC7479243; doi:10.3389/fonc.2020.01682)
Supplement: TABLE S2 — Abundance of F. nucleatum (species level). [file Table_2.docx]

|  | **Normal** | **Tumour** |
| --- | --- | --- |
| **Case A** | **0** | **0.002366737** |
| **Case B** | **0.00528806** | **0.04955309** |
| **Case C** | **0.01511317** | **0.02424498** |
| **Case D** | **0.03492704** | **0.1338809** |
| **Case E** | **0.0651833** | **0.00171135** |
| **Case F** | **0.000114164** | **0.01730487** |
| **Case G** | **0.05193477** | **0.3404964** |
| **Case H** | **0.2069137** | **0.3853866** |
| **Case I** | **0.3935222** | **2.078268** |
| **Case J** | **0.04956049** | **2.831193** |

**Supplementary Table 2: Abundance of *F. nucleatum* (species level)**
